# Effects of Hemodialysis on Intraocular Pressure and Ocular Biological Parameters in Different Angle Structures

**DOI:** 10.1155/2022/9261653

**Published:** 2022-02-12

**Authors:** Fenglei Wang, Ling Wang, Zhiying Yu, Nan Chen, Dabo Wang

**Affiliations:** Ophthalmology, The Affiliated Hospital of Qingdao University, 1677, Wutaishan Road, 266000 Huangdao Region, Qingdao, China

## Abstract

**Purpose:**

This study is aimed at evaluating the effects of hemodialysis on intraocular pressure (IOP) and exploring the possible factors affecting IOP.

**Methods:**

Fifty-two patients with hemodialysis (HD) that were diagnosed with chronic renal failure by nephrology were divided into four groups: wide angle, narrow angle, extremely narrow angle, and closed angle. IOP, central anterior chamber depth (ACD), lens thickness (LT), angle opening distance (AOD), trabecular-iris angle (TIA), iris thickness (IT), and ciliary body thickness (CBT) were recorded before and after HD. The Pearson coefficient test was used to determine correlations among changes in IOP and AOD, ACD, TIA, IT, CBT, and LT.

**Results:**

The IOP in the extremely narrow angle group had significant difference compared with that in the wide angle group and narrow angle group (*P* < 0.05, *P* < 0.01). In the narrow angle group, change in LT was positively correlated with change in IOP (*P* < 0.05). In the extremely narrow angle group, change in LT was positively correlated with change in IOP (P<0.01), whereas changes in AOD and TIA were negatively correlated with change in IOP (*P* < 0.01; *P* < 0.05).

**Conclusion:**

The effect of HD on IOP varies with the structure of the anterior chamber. The increasing of IOP in the extremely narrow-angle group is related with the changes of structure of anterior chamber.

## 1. Introduction

Hemodialysis is one of the renal replacement therapy methods used in patients with chronic renal failure. It involves the exchange of substances via diffusion or convection, which removes metabolic waste products from the body and corrects the water electrolyte balance. However, some patients may experience symptoms such as blurred vision, eye pain, and headache during or after dialysis [1, 2]. Whether elevated intraocular pressure during long-term dialysis induces glaucoma or other ocular complications in patients on chronic maintenance hemodialysis has attracted the attention of ophthalmologists and prompted relevant research.

In some studies, an elevated IOP was measured after hemodialysis [3, 4], while others found no significant changes or even a decreased IOP [5–8]. The reasons for these differences are unclear and may be related to the patient selection criteria utilized and factors influencing changes in IOP. There is currently little evidence available to draw upon to clarify whether this is related to the configuration of the anterior chamber angle.

It is known that chronic renal failure patients have insufficient local blood supply to the eyes [5], and fluctuations in IOP caused by hemodialysis may aggravate this condition or even cause severe, irreversible ischemic, and hypoxic damage to the optic nerve and retina. Therefore, it is necessary to analyze changes in IOP during hemodialysis and investigate related mechanisms to protect visual function in maintenance hemodialysis patients. The primary aims of the current study were to identify parameters associated with a high risk of visual function endangerment in maintenance hemodialysis patients.

## 2. Materials and Methods

### 2.1. Materials

#### 2.1.1. Study Population

Fifty-two eyes in 52 patients (25 male and 27 female) diagnosed with chronic renal failure and undergoing maintenance hemodialysis in the nephrology department of our hospital from January to December 2015 were enrolled in the current study. Informed consent was obtained from all individual participants included in the study. General characteristics of patients are summarized in Table 1. Only the right eyes of the patients were examined.

#### 2.1.2. Inclusion and Exclusion Criteria

Inclusion criteria were as follows:

(1) Patients who exhibited good compliance and appropriate levels of physical activity (2-5 hours per week, such as walking, jogging, etc.) and had signed informed consents, (2) patients whose visual acuity was ≥0.3 on the LogMAR scale, and (3) patients whose IOP was 10–21 mmHg

Exclusion criteria were as follows:

1. Patients with a history of glaucoma or ocular hypertension or eye surgery, (2) patients suffering from concomitant vitreoretinal disorders, (3) patients exhibiting media opacity that affects optical clarity, and (4) patients diagnosed with diabetes

Patients were allocated to one of four groups based on the Shaffer grading system [9], which considers the width of the angle formed by the imaginary tangents of the cornea-trabecular meshwork and the anterior surface of the iris (i.e., the angle of the iris recess serves as the classification criterion) and ultrasound biomicroscopy examinations of which 3 or more quadrants met the reference standards. More specifically, patients with 3 or 4 quadrants with an open angle of >20 degrees were allocated to the wide-angle group (*n* = 22), patients with 3 or 4 quadrants with an open angle of 11–20 degrees were allocated to the narrow-angle group (*n* = 18), patients with 3 or 4 quadrants with an open angle of 0-10 degrees were allocated to the extremely narrow-angle group (*n* = 12), and patients with 3 or 4 quadrants with an open angle of 0 degrees were allocated to the closed-angle group (*n* = 0).

### 2.2. Methods

Hemodialysis patients were allocated to a wide-angle group (WA group), a narrow-angle group (NA group), an extremely narrow-angle group (ENA group), or a closed-angle group (CA group) based on their angular anatomy. Changes in IOP before and after HD were monitored in each group. Changes in anterior chamber parameters were investigated, and correlations between changes in IOP and changes in anterior chamber parameters were assessed.

#### 2.2.1. Hemodialysis Method

Only morning session HD patients were included. All patients underwent 4 h HD sessions, 3 days per week, at a blood-flow rate of 250 mL/min. Patients were treated using high-performance dialyzers: 4008S-type Fresenius HD machine (Germany) and a Campbell 8 L reusable dialyzer (Sweden); patient blood was dialyzed against bicarbonate dialysate (1.5 mmol/L calcium). All patients exhibited arteriovenous fistulae and used a polysulfone hollow-fiber dialyzer (Fx80; Germany). The total body weight was measured before and after HD.

#### 2.2.2. Measurement of Plasma Osmotic Pressure

Blood samples were obtained within 60 seconds before the start and end of HD and then used to calculate plasma osmotic pressure (plasma osmotic pressure = 1.86 × Na + glucose ÷ 18 + urea nitrogen ÷ 2 + 9).

#### 2.2.3. Measurement of IOP

IOP was measured 30 min before the start of HD, 2 h after initiation of HD, and 30 min after ending HD, using a hand-held rebound tonometer (Suowei Rebound tonometer SW-500, Tianjin, China). Three consecutive measurements were averaged to obtain a mean IOP value.

#### 2.2.4. A-Ultrasound and Ultrasound Biomicroscopy (UBM) Measurements

Patients were placed in the supine position. After administration of a topical anesthetic (0.25% oxybuprocaine eye drop), the patients were allowed to fix their fingers to maintain vision straight ahead. Then, central anterior chamber depth and lens thickness were measured by A-ultrasonography (Quantel Medical, Ltd., Model Aviso, France), 30 min before HD and after ending HD. Ten measurements were performed, and the average value was used.

**Figure 1:**
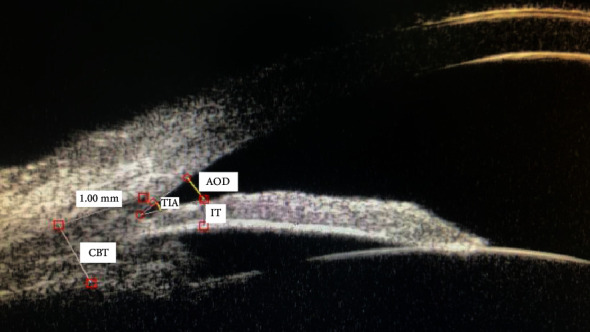
The measurements of anterior chamber parameters by ultrasound biomicroscopy. (AOD was 0.320 mm, TIA was 25.0D, IT was 0.35 mm, and CBT was 0.81 mm.)

Patients were placed in the supine position, with constant indoor brightness. The UBM examinations were performed by one trained technician who used the UBM (Suowei Panoramic Biological Microscope SW-3200 L, Tianjin, China) with a 50 MHz transducer probe. A total of 30 min before the start and end of hemodialysis, angle opening distance (AOD), trabecular-iris angle (TIA), iris thickness (IT), and ciliary body thickness (CBT) were measured by UBM at 12 o'clock, 6 o'clock, 3 o'clock, and 9 o'clock positions. All measurements were performed by the same examiner to minimize the bias, and the average values of the four quadrants were used:

1. AOD: a straight line was drawn perpendicular to the corneal endothelium 500 *μ*m anterior to the scleral spur to the plane of the trabecular mesh, to the corresponding frontal surface of the iris. The length of this line was deemed to be the angle opening distance (Figure 1)
2. TIA: a triangle with the AOD as a base and the recess at the iris root as a vertex and the angle between the vertices was the trabecular-iris angle (Figure 1)
3. IT: the iris thickness was traced at a specific point. The exact location was determined tracing a straight line perpendicular to the trabecular meshwork, 500 *μ*m in front of the scleral spur, which intersect the anterior surface of the iris (Figure 1)
4. CBT: the thickness of ciliary body at the posterior 1 mm of the scleral spur (Figure 1)

### 2.3. Statistical Analysis

All data were analyzed using the SPSS statistical package, version 19.0. The Kolmogorov-Smirnov test was used to assess normality of the data. Variables comparisons in each group were performed utilizing the paired-sample *t*-test. IOP among groups was compared using the repeated measures one-way analysis of variance (ANOVA). The Pearson coefficient test was used to determine correlations among changes in IOP and AOD, ACD, TIA, IT, CBT, and LT. *P* < 0.05 was considered to be statistically significant.

## 3. Results

### 3.1. Change in Plasma Osmotic Pressure

Plasma osmotic pressure after HD was significantly lower than plasma osmotic pressure before HD (*P* < 0.05, *t* = 3.041), as shown in Table 2.

### 3.2. Change in IOP

Changes in IOP in each group 2 h after the initiation of hemodialysis and 30 min after the end of hemodialysis are shown in Table 3.

### 3.3. The Main Effect of Treatment and Time

**Figure 2:**
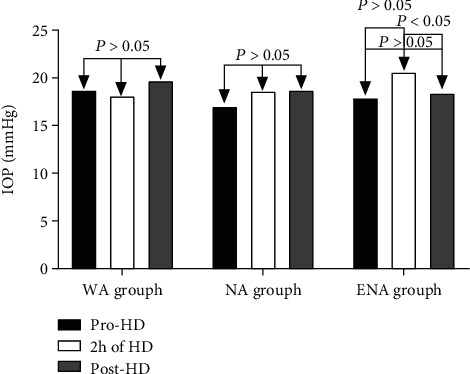
Intraocular pressure before hemodialysis, after 2 h of dialysis, and 30 min after the end of dialysis in three groups.

1. Mean IOP differed significantly at different time points during hemodialysis (*F* = 41.69, *P* < 0.01). The difference between IOP before hemodialysis and 2 h after the initiation of hemodialysis was statistically significant (*P* < 0.01), as was the difference between IOP 2 h after hemodialysis initiation and 30 min after the end of hemodialysis (*P* < 0.01)
2. Mean IOP differed significantly in different groups of hemodialysis patients (*F* = 6.44, *P* < 0.01). IOP in the wide-angle group differed significantly from that in the extremely narrow-angle group (*P* < 0.05), and IOP in the narrow-angle group differed significantly from that in the extremely narrow-angle group (*P* < 0.01) (Table 3)
3. The extremely narrow-angle group had significantly increased IOP 2 h after the initiation of hemodialysis (*P* < 0.05), and in that group IOP returned to prehemodialysis levels within 30 min after the end of hemodialysis (*P* > 0.05) (Figure 2)

### 3.4. Correlations between Changes in Anterior Chamber Parameters and IOP

The ciliary body was thinner in all groups within 30 min after the end of hemodialysis, but it was only statistically significantly thinner than before hemodialysis in the wide-angle group (*t* = 2.61, *P* < 0.05). After hemodialysis, central anterior chamber depth, angle opening distance, and trabecular-iris angle were all smaller in all groups. The root of the iris became thinner, and the lens became thicker, but these differences were not statistically significant (*P* > 0.05) (Tables 4–6).

**Figure 3:**
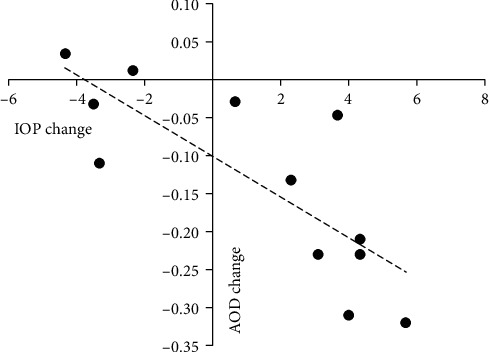
Change in AOD plotted against change in IOP in the extremely narrow-angle group. A significant correlation was found (*r* = −0.845, *P* = 0.004).

**Figure 4:**
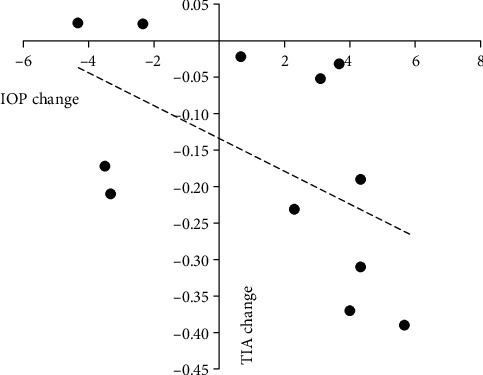
Change in TIA plotted against change in IOP in the extremely narrow-angle group. A significant correlation was found (*r* = −0.753, *P* = 0.019).

**Figure 5:**
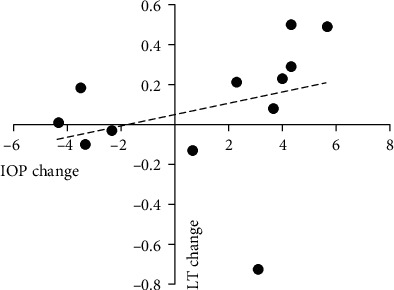
Change in LT plotted against change in IOP in the extremely narrow-angle group. A significant correlation was found (*r* = 0.820, *P* = 0.007).

In the wide-angle group, there were no significant correlations between changes in anterior chamber parameters and the change in IOP. In the narrow-angle group, the change in lens thickness was positively correlated with the change in IOP. In the extremely narrow-angle group, the change in lens thickness was positively correlated with the change in IOP, whereas the changes in AOD and TIA were negatively correlated with the change in IOP (Tables 4–6; Figures 3–5).

## 4. Discussion

The effects of hemodialysis on IOP have been studied by many researchers in recent years. There are many different views on the relationship between hemodialysis and IOP, probably due to differences in research methods, hemodialysis parameters, selected subjects, and measuring time. Most researchers have observed increased IOP after hemodialysis. In 1964, Sitprija et al. [10, 11] reported the earliest study investigating relationships between hemodialysis and changes in IOP. They reported that hemodialysis could lead to an increase in IOP in animal models and uremia patients, with an average increase of 4-8 mmHg. They also reported that IOP increased by an average of 5.9 mmHg in the first 3 hours of hemodialysis. In a study by De Marchi et al. [12], IOP increased significantly in 10 patients with narrow anterior chamber angles during hemodialysis (7.8-12.5 mmHg). Some researchers have reported that IOP did not change over the entire duration of hemodialysis. In a study by Hojs and Pahor [13], there was no statistically significant difference between IOP before and after hemodialysis. A small number of researchers have reported reductions in IOP after hemodialysis. Gutmann and Vaziri [14] investigated IOP in hemodialysis patients and normal controls and reported that IOP was significantly reduced in hemodialysis patients, especially during the first 2 hours, but there was a slight increase at the end of hemodialysis. In a study by Dinc et al. [15] involving 33 patients on hemodialysis, IOP decreased by approximately 1.3 mmHg after hemodialysis. Yang et al. [16] investigated changes in IOP after hemodialysis in 34 patients with chronic renal failure and reported that mean IOP before hemodialysis was 15.1 ± 2.6 mmHg, and it decreased to 13.9 ± 2.2 mmHg after hemodialysis. Cecchin et al. [4] found patients with elevated IOP after hemodialysis have a narrow anterior chamber angle, but there have been no relevant studies on changes in this narrow angle.

In the current study, IOP was significantly increased after 2 hours of hemodialysis in the extremely narrow-angle group, whereas in the wide-angle group and the narrow-angle group, IOP was not significantly increased after 2 hours of hemodialysis. Our results suggest that there may be a dichotomous explanation for the IOP fluctuations. During the dialysis process, toxic substances in the blood (including urea, nitrogen, and creatinine) diffuse into the dialysate, leading to a significant decrease in plasma osmotic pressure [17–19], just as our study showed. And this will increase the aqueous humor production, which can increase IOP. Urea nitrogen concentration in the lens cannot decrease as rapidly as in blood during hemodialysis, resulting in an osmotic pressure imbalance between the lens and the aqueous humor. The lens absorbs water and expands, along with a reduced anterior chamber depth and a narrow angle leading to a poor outflow, and hence IOP also increases [20, 21]. The counterpart is that dialysis can induce dehydration and ultrafiltration, leading to a thinning of the iris and ciliary body with widening the anterior chamber [22]. Consequently, increasing aqueous humor drainage can reduce IOP. Thus, there are many factors that can increase and decrease IOP during hemodialysis.

In this study, despite the increases in aqueous humor production in the wide-angle and narrow-angle groups, the compensatory capacity of the aqueous humor drainage pathway was relatively high, aqueous humor production and drainage were in equilibrium, and fluctuations in IOP during and after hemodialysis were not significant. However, aqueous humor drainage is low in patients with a shallow anterior chamber and a narrow anterior chamber angle [23, 24]. As plasma osmotic pressure decreases after hemodialysis, increased aqueous humor secretion may lead to an increase in IOP and may even cause acute angle-closure glaucoma. Therefore, compared with the other groups, the IOP of 2 hours after initiation of HD was significantly higher than that before hemodialysis in the extremely narrow-angle group.

Notably, 30 min after the end of hemodialysis, IOP had returned to prehemodialysis levels, even in the extremely narrow-angle group. Because in this group, the chamber angle was not completely closed, and the observed fluctuations in IOP during hemodialysis could recover after hemodialysis. An acute increase in IOP is highly likely in patients with complete angle closure [23]. There were no patients with complete angle closure among the 52 cases in the study, which may explain why no acute increases in IOP during hemodialysis were observed. Therefore, we believe that the change in IOP during hemodialysis in patients with chronic renal failure is related to anterior chamber angle structure. An extremely narrow angle is a risk factor for elevated IOP during hemodialysis, while wide-angle patients are relatively safe. Our findings confirmed the importance of anterior chamber status by analyzing the relationship between anterior chamber parameters and IOP changes after hemodialysis. In the extremely narrow-angle group, lens thickening was associated with increasing IOP, whereas changes in AOD and TIA were negatively correlated with IOP values. It is conceivable that osmotic pressure differences caused by hemodialysis may increase lens thickness. The lens thickening leads to a smaller angle opening distance, a narrower trabecular iris angle, and thus increased resistance to aqueous humor drainage. These anatomical modifications are more pronounced in patients with narrow angles.

In recent years, some researchers have also investigated the effects of hemodialysis on anterior chamber parameters. Chong et al. [25] observed changes in anterior chamber parameters before and 2 hours after the end of peritoneal dialysis, including anterior chamber depth, anterior chamber width, anterior chamber area, anterior chamber volume, lens vault, angle opening distance, trabecular iris space area, and angle recess area. Importantly, they reported that there were no statistically significant changes in any of the parameters after dialysis. Caglayan et al. [26] investigated anterior chamber parameters including anterior chamber depth (ACD), aqueous depth (AQD), anterior chamber volume (ACV), and anterior chamber angle (ACA) in 50 eyes before and 30 min after hemodialysis and reported that there were no significant changes in any of these parameters. This is consistent with the changes in anterior chamber parameters before and after hemodialysis in the present study. In another study, changes in anterior chamber depth were investigated between baseline (the beginning of hemodialysis) and 2 and 4 hours after the start of hemodialysis. In that study, anterior chamber depth was only significantly decreased 2 hours after the start of dialysis [27].

It is known that chronic renal failure patients have insufficient local blood supply to the eyes, and fluctuations in IOP caused by hemodialysis may aggravate this condition or even cause severe irreversible ischemic and hypoxic damage to the optic nerve and retina [28]. Due to the limited anterior chamber angle function of the relevant patients, although the increases in IOP in the extremely narrow-angle group were not sufficient to cause acute high IOP, fluctuation in IOP may affect blood supply to the eye in this particular group of patients with chronic renal failure. In addition, among patients on hemodialysis, there are some with even narrower angles than were observed in the present study and some with complete angle closure. Therefore, it is necessary to improve the screening of high-risk groups before hemodialysis. For example, in patients with cataracts in the initial expansion stage, the lens absorbs water and expands during hemodialysis due to the effects of the osmotic gradient, causing the lens and iris to move forward [20, 21], which may lead to relative pupillary block. Aqueous humor drainage is blocked, and IOP increases. In addition, patients with closed-angle glaucoma anatomy (such as small eyeball, shallow anterior chamber, and narrow anterior chamber) have drainage problems that lead to increased IOP. In such patients, effective preventive measures such as early cataract surgery or preventive laser peripheral iridotomy should be considered before hemodialysis. High levels of dehydration should also be prevented during hemodialysis. This not only ensures sufficient hemodialysis but also maintains a relatively stable internal environment after each treatment, thereby reducing the occurrence of ocular complications.

In this study, we observed changes in IOP before, during, and after hemodialysis in each group of patients. With respect to anterior chamber parameters, we did not conduct dynamic observation during hemodialysis and thus did not collect anterior chamber-related data when IOP reached its peak, due to procedural limitations, the relative cleanliness of the environment of the dialysis room, the degree of patient cooperation, and the configuration of the equipment, among other reasons. These parameters may recover very rapidly after hemodialysis; so, although they tend to decrease or increase at the end of hemodialysis, the differences are not statistically significant. This does not mean that these relevant parameters did not change during dialysis. The change in IOP after the end of hemodialysis indicates that the increased IOP during hemodialysis in the extremely narrow-angle group had returned to prehemodialysis levels at the end of hemodialysis. Possibly because of the small sample size, there were no cases of complete angle closure in the present study. In future studies, the sample size will be increased, and dynamic changes in anterior parameters during the dialysis process will be investigated to the greatest extent possible.

## Figures and Tables

**Table 1:** General characteristics of the patients.

Variable	Mean	SD	Range
Age (years)	52.4	10.1	40-68
Hemodialysis duration (year)	6.1	1.9	4-10
Total amount of ultrafiltration (mL)	2318	612.3	1500-3100
Weight loss (kg)	2.10	0.91	0.95-2.95

**Table 2:** Effect of hemodialysis on plasma osmotic pressure and blood biochemical parameters ($\bar{x}\pm s$).

Variable	Pre-HD	Post-HD
Urea nitrogen (mmol/l)	26.06 ± 5.48	8.92 ± 3.79
Glucose (mmol/l)	5.96 ± 1.19	7.85 ± 3.25
Na+ (mmol/l)	138.18 ± 2.40	137.98 ± 4.35
Creatinine (*μ*mol/l)	909.22 ± 119.93	395.52 ± 118.92
Plasma osmotic pressure (mOsm/kgH_2_O)	279.37 ± 6.16	270.54 ± 7.32

**Table 3:** Intraocular pressure before hemodialysis, after 2 h of dialysis, and 30 min after the end of dialysis ($\bar{x}\pm s$, mmHg).

Group	*n*	Pre-HD	2 h of HD	Post-HD
WA group	22	18.7 ± 3.8	18.1 ± 4.9	19.7 ± 5.4
NA group	18	17.0 ± 4.4	18.6 ± 5.7	18.7 ± 6.1
ENA group	12	17.0 ± 4.7	20.6 ± 4.0	18.4 ± 3.8

**Table 4:** Changes in anterior chamber parameters after hemodialysis in the wide-angle group and correlations with changes in IOP.

Variable	Pre-HD	Post-HD	*P* value	Correlation^∗^	
				*r*	*P* value
IOP (mmHg)	18.7 ± 3.8	19.7 ± 5.4	0.224		
AOD (mm)	0.325 ± 0.077	0.321 ± 0.073	0.609	0.186	0.506
ACD (mm)	2.87 ± 0.31	2.84 ± 0.26	0.415	-0.008	0.976
TIA (D)	27.86 ± 5.78	27.55 ± 5.71	0.697	0.466	0.080
IT (mm)	0.425 ± 0.066	0.367 ± 0.055	0.393	0.017	0.952
CBT (mm)	0.707 ± 0.040	0.688 ± 0.044	0.021	0.129	0.647
LT (mm)	4.85 ± 0.33	4.86 ± 0.42	0.816	-0.296	0.285

^∗^Correlation between changes in intraocular pressure and other variables.

**Table 5:** Changes in anterior chamber parameters after hemodialysis in the narrow-angle group and correlations with changes in IOP.

Variable	Pre-HD	Post-HD	*P* value	Correlation^∗^	
				*r*	*P* value
IOP (mmHg)	17.0 ± 4.4	18.7 ± 6.1	0.250		
AOD (mm)	0.225 ± 0.052	0.206 ± 0.058	0.144	-0.057	0.853
ACD (mm)	2.69 ± 0.24	2.59 ± 0.27	0.051	-0.049	0.874
TIA (D)	16.84 ± 3.08	15.22 ± 3.66	0.150	-0.361	0.226
IT (mm)	0.353 ± 0.056	0.350 ± 0.052	0.494	-0.282	0.351
CBT (mm)	0.669 ± 0.064	0.659 ± 0.067	0.218	0.444	0.129
LT (mm)	4.96 ± 0.53	5.04 ± 0.52	0.121	0.610	0.027

^∗^Correlation between changes in intraocular pressure and other variables.

**Table 6:** Changes in anterior chamber parameters after hemodialysis in the extremely narrow-angle group and correlations with changes in IOP.

Variable	Pre-HD	Post-HD	*P* value	Correlation^∗^	
				*r*	*P* value
IOP (mmHg)	17.0 ± 4.7	18.4 ± 3.8	0.302		
AOD (mm)	0.134 ± 0.064	0.124 ± 0.060	0.532	-0.845	0.004
ACD (mm)	2.65 ± 0.15	2.59 ± 0.16	0.338	-0.130	0.738
TIA (D)	8.09 ± 1.55	7.52 ± 1.67	0.705	-0.753	0.019
IT (mm)	0.425 ± 0.066	0.411 ± 0.070	0.293	0.388	0.302
CBT (mm)	0.669 ± 0.064	0.648 ± 0.057	0.088	-0.218	0.574
LT (mm)	4.72 ± 0.31	4.78 ± 0.28	0.334	0.820	0.007

^∗^Correlation between changes in intraocular pressure and other variables.

## Data Availability

The datasets used and/or analyzed during the current study are available from the corresponding author on reasonable request.

## Abbreviations

IOP: Intraocular pressure
HD: Hemodialysis
UBM: Ultrasound biomicroscopy
ACD: Central anterior chamber depth
AOD: Angle opening distance at 500 *μ*m from scleral spur
TIA: Trabecular iris angle
LT: Lens thickness
IT: Iris thickness
CBT: Ciliary body thickness.
